# Genome-wide investigation and expression analysis of Sodium/Calcium exchanger gene family in rice and Arabidopsis

**DOI:** 10.1186/s12284-015-0054-5

**Published:** 2015-07-02

**Authors:** Anil Kumar Singh, Ritesh Kumar, Amit K. Tripathi, Brijesh K. Gupta, Ashwani Pareek, Sneh L. Singla-Pareek

**Affiliations:** Plant Molecular Biology Group, International Centre for Genetic Engineering & Biotechnology (ICGEB), Aruna Asaf Ali Marg, New Delhi, 110067 India; Stress Physiology and Molecular Biology Laboratory, School of Life Sciences, Jawaharlal Nehru University, New Delhi, 110067 India; Present address: Division of Biotechnology, CSIR-Institute of Himalayan Bioresource Technology, Palampur -, 176061, H.P. India; Present address: Division of Applied Life Science, Plant Molecular Biology and Biotechnology Research Center, Gyeongsang National University, Jinju, 660-701 Korea

**Keywords:** Sodium calcium exchanger, Abiotic stress, Development, MPSS, Microarray, qRT-PCR

## Abstract

**Background:**

The Na^+^/Ca^2+^ Exchanger (NCX) protein family is a member of the Cation/Ca^2+^ exchanger superfamily and its members play important roles in cellular Ca^2+^ homeostasis. While the functions of NCX family of proteins is well understood in humans, not much is known about the total complement of Na^+^/Ca^2+^ exchangers in plants and their role in various physiological and developmental processes. In the present study, we have identified all the NCX proteins encoded in the genomes of rice and Arabidopsis and studied their phylogeny, domain architecture and expression profiles across different tissues, at various developmental stages and under stress conditions.

**Results:**

Through whole genome investigation, we identified twenty-two NCX proteins encoded by fifteen genes in rice and sixteen NCX proteins encoded by thirteen genes in Arabidopsis. Based on phylogenetic reconstruction, these could be classified into five clades, members of most of which were found to possess distinct domain architecture. Expression profiling of the identified NCX genes using publicly available MPSS and microarray data showed differential expression patterns under abiotic stresses, and at various development stages. In rice, OsNCX1, OsNCX8, OsNCX9 and OsNCX15 were found to be highly expressed in all the plant parts and various developmental stages. qRT-PCR based expression analysis revealed that OsNCX3, OsNCX10 and OsNCX15 were highly induced by salt and dehydration stress. Besides, expression profiling showed differential regulation of rice NCX genes in response to calcium and EGTA. Interestingly, expression of none of the NCX genes was found to be co-regulated by NaCl and calcium.

**Conclusions:**

Together, our results present insights into the potential role of NCX family of proteins in abiotic stresses and development. Findings of the present investigation should serve as a starting point for future studies aiming functional characterization of plant NCX family proteins.

**Electronic supplementary material:**

The online version of this article (doi:10.1186/s12284-015-0054-5) contains supplementary material, which is available to authorized users.

## Background

Systemic and cellular homeostasis is very important for proper functioning of living organisms, including plants. The alkali (Na^+^, K^+^) and alkaline (Ca^2+^, Mg^2+^) earth metals play very critical roles in living system, but their optimum cellular concentration must be maintained for proper action (Jon et al. 2008). Calcium is one of the most important and ubiquitous signaling molecules and perturbed Ca^2+^ homeostasis may induce cellular death (McLean et al. 1965). The role of Ca^2+^ as a secondary messenger in signal transduction in response to external and internal stimuli like plant growth nutrients, light, fungal elicitors and various abiotic stresses is well known (Hepler and Wayne 1985; Evans et al. 2001). Intracellular Ca^2+^ concentration in resting cells is maintained at 10–100 nM and it may go up to 10 μM during external or internal perturbations (Peretz et al. 1994; Bootman and Berridge 1995; Hardie 1996). As Ca^2+^ controls a number of physiological processes both at molecular and cellular levels in plants (Hepler and Wayne 1985), it is very crucial to regulate the Ca^2+^ level inside the cell.

There are two classes of membrane proteins involved in cellular Ca^2+^ homeostasis, the ATP-driven calcium pumps or plasma membrane Ca^2+^ ATPase (PMCA), and sodium/calcium exchangers (Na^+^/Ca^2+^ exchanger or NCX) (Haug-Collet et al. 1999). These two pumps complement each other in their functions. Though NCX has a low affinity towards Ca^2+^, it can transport Ca^2+^ at very high speed within short duration i.e. up to 5000 calcium ions per second (Carafoli et al. 2001). On the other hand, PMCA has very high affinity towards Ca^2+^, which normally helps the cell in maintaining the very low concentration of cytosolic Ca^2+^ in resting cell (Siegel et al. 1999).

NCXs are antiporter membrane proteins and maintain optimum cellular Ca^2+^ levels. The energy stored in the electrochemical gradient of such proteins is used to exchange Ca^2+^ with its counterpart Na^+^, which moves down its electrochemical gradient across the plasma membrane. NCX is considered as one of the most important cellular component for removing Ca^2+^ (Dipolo and Beauge 2006). NCX functions on the principle of removal of a single calcium ion and import of three sodium ions in exchange (Yu and Choi 1997). NCX also show reversibility in their mode of action i.e. when cellular Na^+^ concentration increases beyond critical level these proteins start importing calcium ions inside the cell (Bindokas and Miller 1995; Yu and Choi 1997; Wolf et al. 2001). Therefore, NCX can work in both directions depending upon the gradient generated by Ca^2+^ and Na^+^ concentrations inside the cell. NCX proteins have been found in many different cell types of animal and plant species. Wide ranges of animal and plant species have highly conserved structural properties of NCX. Amongst animals, invertebrates possess only single NCX gene, whereas vertebrates have multiple NCX genes due to gene duplication (On et al. 2008).

The availability of high quality whole genome sequence of Arabidopsis and rice and their expression data in the form of Microarray and MPSS (Massively Parallel Signature Sequencing) has enabled the study of different gene families which play important roles in plants. Different gene families in Arabidopsis and rice genomes, such as Cation/H^*+*^ exchanger family (Kamiya et al. 2005), histidine kinase family (Pareek et al. 2006), SAUR gene family (Jain et al. 2006), protein phosphatase 2C family (Xue et al. 2008), basic leucine zipper transcription factor family in rice (Nijhawan et al. 2008), CBS domain containing gene family (Kushwaha et al. 2009), nucleocytoplasmic transport receptors family (Huang et al. 2010) and glyoxalase family (Mustafiz et al. 2011) etc. have been analyzed. Recently genome wide analysis of gene families has been extended to other plant species like NAC transcription factor family in potato (Singh et al. 2013), Family‑1 UDP glycosyltransferases in maize (Li et al. 2014), and AP2/ERF family genes from *Lotus corniculatus* (Sun et al. 2014).

Some of the NCX genes are well studied in animal and human systems. Phylogenetic analysis of NCX gene family in fish species has identified 13 NCX genes and revealed that two serial NCX gene duplication events have occurred around the time vertebrates and invertebrates diverged (Marshall et al. 2005). In case of plants, the only characterized NCX protein is AtNCL (an NCX-like protein) from Arabidopsis which was found to play a role in salt stress and maintaining Ca^2+^ homeostasis (Wang et al. 2012). However, other members of the NCX gene family in plants have not been comprehensively identified and characterized. Considering the importance of this protein family in maintaining Ca^2+^-homeostasis and the pivotal roles that Ca^2+^ plays as a second messenger in plants, it is imperative to comprehensively dissect the complement of sodium-calcium exchangers in plants paving the way for functional characterization studies.

Therefore, to gain insights into the structural and functional attributes of NCX family of proteins in plants, in the present study, we have carried out whole genome analyses to identify NCX domain containing proteins in Arabidopsis and rice genomes using TAIR (The Arabidopsis Information Resource) version 10 and RGAP (Rice Genome Annotation Project) release 7, respectively. We have also suggested nomenclature, and provided chromosomal distribution and phylogenetic analysis of NCX genes in Arabidopsis and rice. For a comprehensive phylogenetic analysis, we have additionally identified NCX proteins from two monocotyledonous plant species, Brachypodium (*Brachypodium distachyon*) and foxtail millet (*Setaria italica*) and two dicotyledonous plant species, poplar (*Populus trichocarpa*) and potato (*Solanum tuberosum*). Detailed *in silico* expression analysis of NCX genes during developmental and stress conditions provided important clue regarding their possible functions. Quantitative real time PCR (qRT-PCR) analysis of fifteen rice NCX genes under six different abiotic stresses viz. salinity, dehydration, heat, cold, oxidative and UV and under exogenous treatment with CaCl_2_ and a Ca^2+^ chelator, EGTA (ethylene glycol tetraacetic acid) was performed.

## Results and discussion

### Identification and nomenclature of NCX proteins in Arabidopsis and rice genomes

Genes encoding NCX proteins were identified in Arabidopsis (TAIR version 10) and rice (TIGR version 7) genomes by employing HMM (Hidden Markov Model) profile retrieved from Pfam database, keyword search and domain search functions. NCX domain (PF01699) containing proteins having putative sodium/calcium exchanger function have been classified as NCX proteins. Our analysis has identified thirteen distinct chromosomal loci encoding for sixteen NCX proteins in Arabidopsis and fifteen chromosomal loci encoding for twenty-two NCX proteins in rice; and hence are reported to undergo alternative splicing. In Arabidopsis, only two genes (*AtNCX5* and *AtNCX6*), whereas in rice, seven genes (*OsNCX1, OsNCX2, OsNCX4, OsNCX5*, *OsNCX7, OsNCX9* and *OsNCX14,*) are reported to undergo alternative splicing. In order to confirm the presence of NCX domain and identification of additional domains, all the NCX proteins were analyzed using Pfam and InterProScan. These analyses confirmed the presence of NCX domain in all the identified proteins. In addition, CAX and EF-hand domains were also found in some of the proteins (Table 1). Previously, some NCX family members have been named ambiguously, which has led to confusion in literature. For example, five Arabidopsis proteins which were named as CAX7 to CAX11 by Maser et al. (2001) were later found to be proteins showing very high sequence similarity with mammalian K^+^-dependent Na^+^/Ca^2+^ antiporter NCKX6 (Shigaki et al. 2006). Thus, to avoid any ambiguity and to maintain uniformity, we have suggested new nomenclature for NCX family members. In case where prior information was present in literature regarding any gene, the old name is given along with the new name (Table 1). ‘At’ and ‘Os’ have been used as prefix for the nomenclature in case of *Arabidopsis thaliana* and *Oryza sativa* respectively, followed by NCX. The genes were numbered according to their chromosomal location on chromosome 1–5 in case of Arabidopsis and on chromosome 1–12 in case of rice and from top to bottom. Similar criteria have been adapted for nomenclature of NAC proteins in soybean (Le et al. 2011) and potato (Singh et al. 2013) and WRKY proteins in maize (Wei et al. 2012). Details of each NCX member are shown in Table 1, representing their locus numbers, CDS and protein length, chromosomal co-ordinates and domain architecture.

**Table 1:** Details of putative NCX proteins encoded in the Arabidopsis and rice genomes. Table enlists NCX genes in Arabidopsis and rice along with their corresponding proteins, their existing nomenclature in TAIR, locus identifiers, CDS, protein length, chromosomal locations and domain architecture

Gene	Protein	Nomenclature in TAIR/RGAP	Locus	CDS length (bp)	Protein length (aa)	Coordinates (5′-3′)	Domain architecture
*Arabidopsis thaliana*							
AtNCX1	AtNCX1	AtCAX11	AT1G08960.1	1248	416	2879642 - 2882231	
AtNCX2	AtNCX2	Sodium/calcium exchanger	AT1G53210.1	1758	586	19844632 - 19847836	
AtNCX3	AtNCX3	AtCCX4	AT1G54115.1	1935	645	20202118 - 20204177	
AtNCX4	AtNCX4	AtCAX6	AT1G55720.1	1449	467	20828118 - 20830640	
AtNCX5	AtNCX5.1	AtCAX5	AT1G55730.1	1326	442	20831052 - 20834519	
	AtNCX5.2		AT1G55730.2	1326	442	20831052-20834512	
AtNCX6	AtNCX6.1	AtCAX1	AT2G38170.1	1392	464	15989083 - 15993278	
	AtNCX6.2		AT2G38170.2	1179	393	15989083 - 15993276	
	AtNCX6.3		AT2G38170.3	1428	476	15990045 - 15993278	
AtNCX7	AtNCX7	AtMHX1	AT2G47600.1	1620	540	19524160 - 19527413	
AtNCX8	AtNCX8	AtCAX2	AT3G13320.1	1326	442	4314529 - 4318351	
AtNCX9	AtNCX9	AtCCX3	AT3G14070.1	1932	644	4661143 - 4663074	
AtNCX10	AtNCX10	AtCAX3	AT3G51860.1	1380	460	19239427 - 19242913	
AtNCX11	AtNCX11	AtCAX4	AT5G01490.1	1365	455	195541 - 198524	
AtNCX12	AtNCX12	AtCAX8	AT5G17850.1	1680	560	5899253 - 5900932	
AtNCX13	AtNCX13	AtCAX7	AT5G17860.1	1713	571	5902394 - 5904380	
*Oryza sativa*							
OsNCX1	OsNCX1.1	Sodium/calcium exchanger	LOC_Os01g11414.1	1755	584	6134202 -6139819	
	OsNCX1.2		LOC_Os01g11414.2	1284	427	6136830-6139189	
OsNCX2	OsNCX2.1	OsCAX1a	LOC_Os01g37690.1	1356	451	21076730 - 21071370	
	OsNCX2.2		LOC_Os01g37690.2	1065	354	21073919-21072230	
OsNCX3	OsNCX3	Sodium/calcium exchanger	LOC_Os02g04630.1	1089	362	2070492 - 2075369	
OsNCX4	OsNCX4.1	Sodium/calcium exchanger	LOC_Os02g14980.1	1728	575	8358769 - 8362960	
	OsNCX4.2		LOC_Os02g14980.2	1281	426	8359318-8362959	
OsNCX5	OsNCX5.1	OsCAX1c	LOC_Os02g21009.1	1353	450	12432287 - 12451812	
	OsNCX5.2		LOC_Os02g21009.2	1353	451	12432287 - 12448557	
OsNCX6	OsNCX6	Sodium/calcium exchanger	LOC_Os03g08230.1	1920	639	4196467 - 4194283	
OsNCX7	OsNCX7.1	Sodium/calcium exchanger	LOC_Os03g27960.1	1317	437	16061184 - 16065169	
	OsNCX7.2		LOC_Os03g27960.2	1314	437	16061151 - 16065169	
OsNCX8	OsNCX8	Sodium/calcium exchanger	LOC_Os03g45370.1	1728	575	25613825 - 25616214	
OsNCX9	OsNCX9.1	OsCAX3	LOC_Os04g55940.1	1254	417	33314630 - 33319375	
	OsNCX9.2		LOC_Os04g55940.2	1254	418	33315199-33319375	
OsNCX10	OsNCX10	OsCAX1b	LOC_Os05g51610.1	1362	453	29593841 - 29588646	
OsNCX11	OsNCX11	Sodium/calcium exchanger	LOC_Os10g30070.1	1794	597	15614210 - 15616127	
OsNCX12	OsNCX12	Sodium/calcium exchanger	LOC_Os11g01580.1	1068	355	335287 – 334094	
OsNCX13	OsNCX13	Sodium/calcium exchanger	LOC_Os11g05070.1	798	265	2220748 - 2217645	
OsNCX14	OsNCX14.1	Sodium/calcium exchanger	LOC_Os11g43860.1	1572	523	26492383 - 26488372	
	OsNCX14.2		LOC_Os11g43860.2	1305	434	26492229 - 26488372	
OsNCX15	OsNCX15	Sodium/calcium exchanger	LOC_Os12g42910.1	1764	587	26669155 - 26671200	

NXC,  EF-hand,  CAX domian

Based on the presence of conserved domains, it was found that in Arabidopsis all the NCX proteins contain a pair of NCX domains, except AtNCX2 (At1g53210) that contains only one NCX domain and one EF-hand domain (Table 1). Whereas in rice, eleven NCX genes (*OsNCX2, OsNCX3, OsNCX5, OsNCX6, OsNCX7, OsNCX8, OsNCX9, OsNCX10, OsNCX11, OsNCX14* and *OsNCX15*) code for proteins with a pair of NCX domains, the rest four genes (*OsNCX1, OsNCX4, OsNCX12* and *OsNCX13*) code for proteins with a single NCX domain. Out of four proteins with a single NCX domain, two (OsNCX1 and OsNCX4) contain additional EF-hand domain. Recently, functional characterization of Arabidopsis NCX-like protein, AtNCL (At1g53210; designated as AtNCX2 in present study) was carried out. AtNCL was found to have the ability to bind Ca^2+^ and its loss of function mutants were less sensitive to salt stress than WT or AtNCL overexpressing lines (Wang et al. 2012). Thus, the presence of EF-hand domain in OsNCX1 and OsNCX4 suggest that these proteins may also bind Ca^2+^ and regulate cellular Na^+^ and Ca^2+^ homeostasis.

Among NCX proteins with a pair of NCX domains, six AtNCXs (AtNCX4, AtNCX5, AtNCX6, AtNCX8, AtNCX10 and AtNCX11) and five OsNCXs (OsNCX2, OsNCX5, OsNCX7, OsNCX9 and OsNCX10) also contain a CAX (Ca^2+^/H^+^ exchanger) domain (TIGR00378). Interestingly, both the NCX domains in these proteins were found to lie within the CAX domain. CAX domain is a dominant feature of Cation/H^+^ exchanger proteins, which transport and regulates Ca^2+^ homeostasis. Previous characterization of Arabidopsis CAX1 and CAX2 showed that these genes encode for high efficiency and low efficiency H^+^/Ca^2+^ exchangers, respectively (Hirschi et al. 1996). Since, these CAX genes were characterized before release of Arabidopsis genome sequence, there appears to be some ambiguity. For example, CAX1 and CAX2 were reported to encode for proteins of 459 and 399 amino acids, respectively. However, in the present study we show that CAX1 and CAX2 (designated as AtNCX6 and AtNCX8, respectively) encode for proteins of 463 and 441 amino acids, respectively. A phylogenetic study of cation transporter families showed that Arabidopsis contains eleven CAX genes named AtCAX1-11 (Mäser et al. 2001). However, later it was shown that AtCAX7 to AtCAX11 have limited amino acid sequence homology with any CAX. In fact, these proteins share a very high sequence similarity with the mammalian K^+^-dependent Na^+^/Ca^2+^ antiporter NCKX6 (Cai and Lytton 2004; Shigaki et al. 2006). Therefore, in the present study all the NCX domain containing proteins were renamed as NCX proteins irrespective of the presence of additional domain(s). The presence of NCX domain along with the CAX domain in these proteins suggests that these proteins may also have dual role as CAX and NCX. To ascertain the relevance of co-occurrence of these domains in a single protein, their functional characterization would be required in future.

### Chromosomal organization of NCX genes

**Fig. 1:**
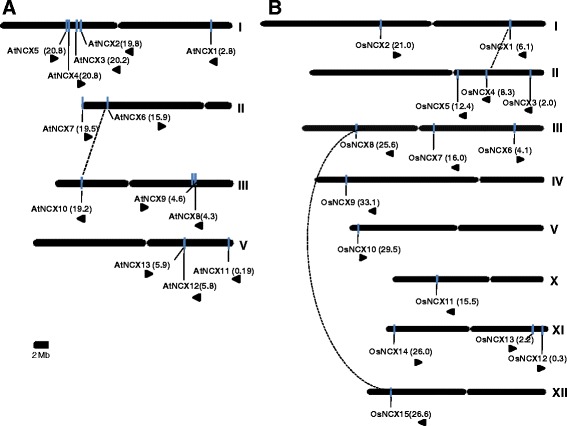
Genomic distribution of NCX genes. **a** Chromosomal localization of NCX family genes from Arabidopsis. Four out of the five Arabidopsis chromosomes (all except chromosome IV) were found to possess one or more gene(s) encoding NCX family proteins. Bars on chromosomes denote the genes and arrows below the gene names indicate the direction of transcription. **b** Genomic distribution of NCX family genes on rice chromosomes. Out of the 12 chromosomes of rice, 8 chromosomes possess genes encoding NCX proteins. Chromosome numbers are shown at the right of the chromosomes and the centromeric regions are indicated by ellipse in both rice and Arabidopsis. Events of duplication between *AtNCX6* and *AtNCX10* in Arabidopsis; and between *OsNCX1* and *OsNCX4* and *OsNCX8* and *OsNCX15* in rice are represented by dotted lines. Scale at the bottom represents 2 Mb for both the karyograms

In case of Arabidopsis, NCX genes are distributed on chromosome I, II, III and V, none of the NCX gene is located on chromosome IV (Fig. 1a). Five NCX genes are located on chromosome I, while two NCX genes are located on chromosome II. Chromosome III and chromosome V possess three NCX genes each. It has been previously reported that two rounds of duplication events have occurred in Arabidopsis genome during the course of evolution, followed by gene loss (Bowers et al. 2003; Ermolaeva et al. 2003). This is evident by the observation that only one NCX gene from Arabidopsis, *AtNCX6* on chromosome II, seems to be duplicated along with inversion on chromosome III as *AtNCX10*. These duplicated genes share more than 75 % homology at protein level (data not shown). Similarly, a few genes of various protein families such as, CDCP (Kushwaha et al. 2009) and glyoxalase (Mustafiz et al. 2011) have been reported to be duplicated in Arabidopsis.

In case of rice, all fifteen NCX genes are distributed on eight out of twelve chromosomes. The chromosome II, III, and XI each possess three NCX genes, while, two NCX genes are located on chromosome I. Rest of the chromosome viz. IV, V, X and XII each contains only single NCX gene (Fig. 1b). Gene duplication plays a central role in plant diversification, a key process that generates the raw material necessary for adaptive evolution (Flagel and Wendel 2009). In case of rice, it has been reported that large-scale losses of duplicated genes might have occurred shortly after the genome duplication (Wang et al. 2005). *OsNCX1* localized on chromosome I seems to be duplicated as *OsNCX4* on chromosome II. These duplicated genes share 65 % sequence homology at protein level (data not shown). Similarly *OsNCX8* on chromosome III seems to be duplicated on chromosome XII as *OsNCX15* and they share 58 % sequence homology at protein level with each other (data not shown).

### Phylogenetic analysis of NCX proteins of Arabidopsis and rice

**Fig. 2:**
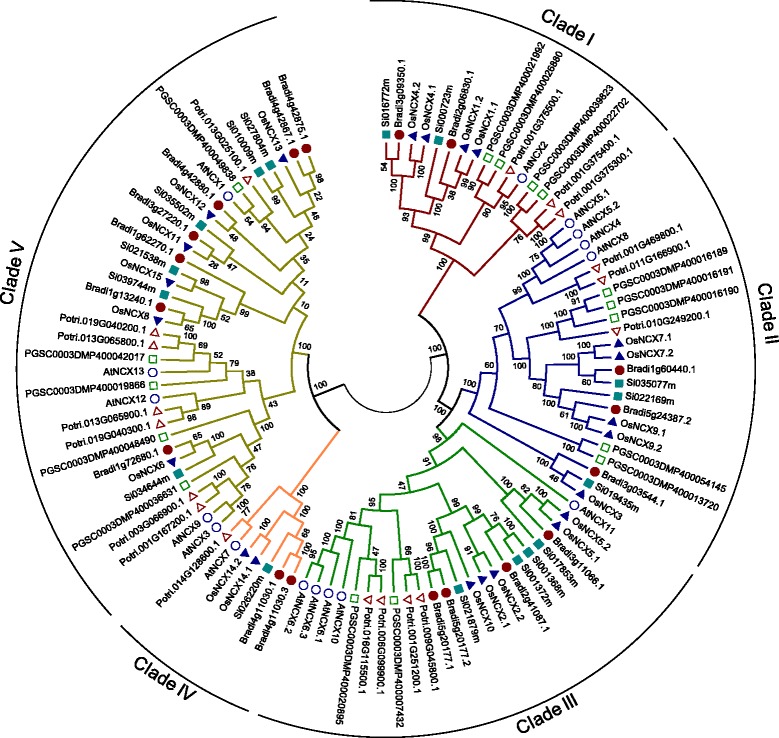
Phylogenetic relationships among Arabidopsis, rice, Brachypodium, foxtail millet, poplar and potato NCX proteins. Multiple sequence alignment of NCX proteins was done using ClustalW2 and the phylogenetic tree was generated using MEGA6 software by the Neighbor-joining method with 1000 bootstrap replicates. NCX proteins were categorized into five different clades depending upon the relative sequence homology of each member of the clades. NCX proteins from Arabidopsis, poplar, potato, rice, *Brachypodium* and foxtail millet are denoted by different shapes viz. circle, triangle, rectangle, solid triangle, solid circle and solid rectangle, respectively

To explore the evolutionary relationship between Arabidopsis and rice NCX proteins, multiple sequence alignment of protein sequences was performed using ClustalW2 program and phylogenetic tree was generated using MEGA6 software (Additional file 1: Figure S1). All the members of Arabidopsis and rice NCX family were divided into five major clades. Interestingly, NCX proteins with similar domain architecture were clustered in same clades. Proteins such as AtNCX2, OsNCX1, and OsNCX4, which have calcium binding EF hand-like domain in addition to NCX domain were clustered together in clade I and share 46 to 58 % sequence homology with each other. In clade II, AtNCX4, AtNCX5, AtNCX8, OsNCX3, OsNCX7 and OsNCX9 were clustered together and share 46 to 87 % sequence homology with each other. All the proteins clustered in clade II contain a CAX domain in addition to a pair of NCX domains, except OsNCX3 that contains only a pair of NCX domains. Clade III is the second largest clade, which contains AtNCX6, AtNCX10, AtNCX11, OsNCX2, OsNCX5 and OsNCX10. All the proteins in this clade contain CAX domain in addition to a pair of NCX domains and share 46 to 76 % sequence homology with each other. The clade IV is the smallest clade, having only AtNCX7 and OsNCX14, which share 67.5 % sequence homology with each other. The largest clade V contains eleven proteins (AtNCX3, AtNCX9, OsNCX6, AtNCX12, AtNCX13, OsNCX8, OsNCX15, OsNCX11, OsNCX12, AtNCX1, and OsNCX13) which share sequence similarity ranging from 42 to 80 %. In order to study the phylogenetic relationship of Arabidopsis and rice NCX family members with that of other plants, NCX proteins were identified from the *Brachypodium* and foxtail millet as representatives of monocotyledonous and from poplar and potato as representatives of dicotyledonous plants. We have identified 18, 16, 18 and 16 NCX proteins from *Brachypodium*, foxtail millet, poplar and potato*,* respectively (Additional file 2: Table S1). Orthologs of rice NCX proteins in *Brachypodium* and foxtail millet and that of Arabidopsis NCX proteins in poplar and potato were also identified (Additional file 2: Table S1). The presence of NCX domain in all these proteins was verified using Pfam and InterProScan. However, in some of the proteins CAX and EF hand domains were also identified (Additional file 3: Table S2). Phylogenetic tree comprising NCX proteins from Arabidopsis and rice with those from *Brachypodium*, foxtail millet, poplar and potato was constructed using MEGA6 (Fig. 2). Interestingly, NCX proteins from all the plant species studied (except potato) were divided into five clades as found in the case of Arabidopsis and rice NCX proteins, shown in Additional file 1: Figure S1. NCX proteins from potato were found to be distributed in four phylogenetic clades (all except clade IV). Clade V consists of maximum number of NCX proteins (36), followed by clade III, II, I and IV, which contain 24, 23, 16 and 7 NCX proteins, respectively. Similar distribution of NCX proteins of all the six species in five clades was found to be strikingly similar (Fig. 2) as in case of Arabidopsis and rice NCXs (Additional file 1: Figure S1), which was found to be based on domain architecture. Cai and Lytton (2004) have previously performed phylogenetic analysis of the cation/Ca^2+^ exchangers super family of 147 proteins from different genomes like bacteria, archaea, and eukaryotes, and classified this super family into the YBRG, CAX, NCX, NCKX and CCX families. Phylogenetic relationship of five CAX genes of rice was suggested earlier by Kamiya et al. (2005) which are part of NCX family also. Recently, Emery et al. 2012 have also suggested the evolutionary hierarchy of Ca^2+^/cation antiporter families in flowering plants which also includes NCX family. The present study provides a comprehensive phylogenetic analysis of all NCX proteins identified from *A. thaliana, O. sativa*, *B. distachyon, S. italica, P. trichocarpa* and *S. tuberosum*.

### Topological model prediction for NCX proteins

**Fig. 3:**
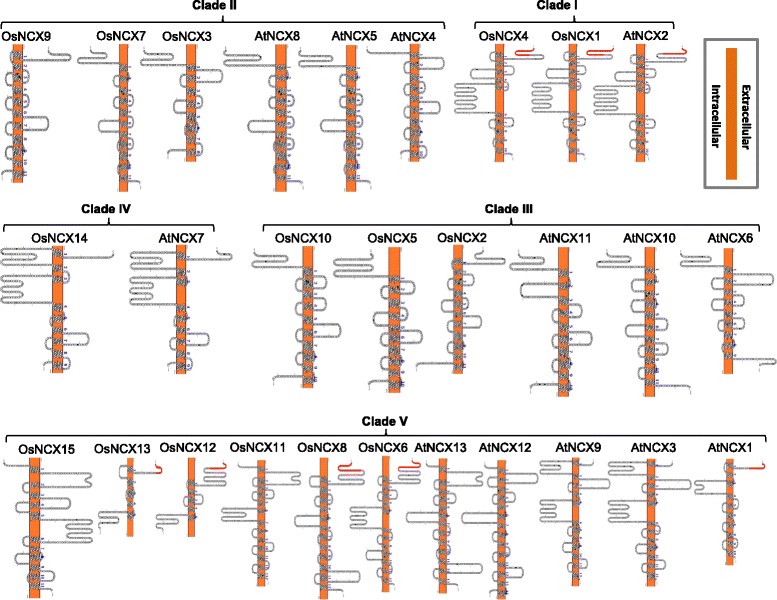
Predicted topological structure of each of the NCX family members in Arabidopsis and rice. Here, all the members are divided into clades according to phylogenetic relationship among Arabidopsis and rice NCX families (as shown in Additional file 1: Figure S1 and in Fig. 3). Topological structures were generated using Protter v1.0 (see Methods). For splice variants possessing a similar predicted topological architecture, only a single model for the topology has been provided. Red colored N-terminal regions in a few NCX proteins represent putative signal peptides. Finger-like projections are loops joining N- and C-terminus groups of TMS, these loops may act as EF hand. The box at the top-left is a schematic representation of the plasma membrane (shown as orange bar) with the extra- and the intra-cellular regions specified

Topological structures of all the NCX proteins of Arabidopsis and rice (for the longest ORFs among the spliced variants) were predicted using Protter software (Fig. 3). Presence of transmembrane segments (TMSs) indicated that all the NCX proteins are transmembrane proteins. In general, proteins clustered in same clade in phylogenetic tree (Additional file 1: Figure S1) share similar membrane topologies. Thus, topological models were arranged as per the phylogenetic clustering of NCX proteins. The number of TMSs was found to be variable in various NCX proteins and ranged from 5 to 14. In many NCX proteins from both rice and Arabidopsis, a large hydrophilic loop intruding into the cytoplasm apparently separated the TMSs into two groups joined together by the large hydrophilic loop. However, as an exception, in case of OsNCX15, the TMSs were found to be separated by an extracellular loop. It was observed that generally lesser number of TMSs lie towards the N-terminal side of the loop and more were present towards C-terminal side (Fig. 3). This loop comprises sites important for calcium regulation, Na^+^ dependent inactivation and alternative splicing (Iwamoto et al. 1999; Marshall et al. 2005). Some proteins such as AtNCX2, OsNCX1 and OsNCX4 also have EF hand loop in between two spans of TMS (marked as clade I in Fig. 3). Two spans of TMS have also been reported earlier in case of NCX domain containing calcium/proton exchangers (CAX), calcium/cation exchangers (CCX) and Magnesium/cation exchangers (MHX) in Arabidopsis, but they differ in function with little modification in the N- and C terminal residues of TMS (Kamiya and Maeshima 2004; Shigaki et al. 2006). Based on experimental evidences, NCX proteins are modeled to have nine putative TMSs (Iwamoto et al. 1999; Nicoll et al. 1999) in animals. Five TMSs were reported to be present in the N-terminal domain and 4 in the C-terminal domain. These NCX proteins comprised ~30 residues long signal peptide that is cleaved during initial processing. Interestingly, proteins with signal peptide on their N-terminal were clustered in clade I and clade V (Fig. 3).

### Expression profiling of NCX family genes using Massive parallel signature sequencing (MPSS)-based expression data

**Fig. 4:**
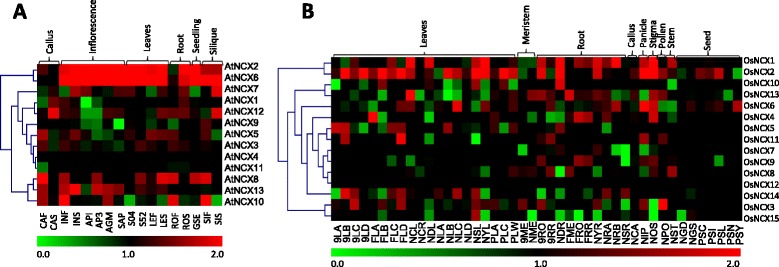
Expression profile of NCX genes as obtained from analysis of MPSS-based expression data. **a** Expression profile of NCX genes from MPSS data in different tissues/organs of Arabidopsis, **b** Expression profile of NCX genes from MPSS data in different tissues/organs in rice. The heat maps have been generated using TIGR MeV software package and represent hierarchical clustering of log-transformed signal values of all NCX genes in various tissue/organs (indicated at the top). The scale for relative expression values is represented by color bars below the heat maps, thereby green color representing lowest expression levels, black medium expression and red denotes highest expression level. Names of the MPSS libraries are mentioned below each heat map

Expression analysis of NCX gene family members using Arabidopsis MPSS data (Fig. 4a) revealed that *AtNCX2* and *AtNCX6* have high transcript level in all the tissues except callus (CAF and CAS) and untreated root tissue (ROF), suggesting their role in most of the development stages. The expression of *AtNCX7* seems to be higher only in inflorescence and silique, and remained lower in all other tissues suggesting its specific role in flowering and silique development. Earlier, characterization of AtMHX (a Mg^2+^/H^+^ exchanger also found to possess NCX domain; named as AtNCX7 in present study) has been done by Shaul et al. (1999) and it was found to have highest expression in inflorescence, which correspond to our findings (Fig. 4a). Similarly, *AtNCX12* has high expression only in case of silique (SIF) and callus (CAS). *AtNCX11* and *AtNCX4* maintain constitutively medium level of expression in almost all the plant tissues. Expression of *AtNCX13* was high in inflorescence tissues. *AtNCX10* exhibited higher transcript level in inflorescence (INF), root (ROF) and silique (SIF) tissues.

Further, we have analyzed the expression pattern of all the rice NCX genes in different tissues using MPSS data (Fig. 4b). Transcript of *OsNCX2* was predominantly high in all the tissues, which possibly indicates its important role throughout the plant. OsNCX12 has uniformly intermediate level of expression in all the plant tissues. Previously, the transcript levels of rice CAX genes i.e. *OsCAX1a* (*OsNCX2*), *OsCAX1b* (*OsNCX10*), *OsCAX1c* (*OsNCX5*), *OsCAX2* (*OsNCX7*) and *OsCAX3* (*OsNCX9*), were analyzed in several tissues like, embryo, endosperm, shoot, root, flower, leaf blade, leaf sheath, node, internode, root and callus (Kamiya et al. 2005). Expression profile of these genes reported earlier corresponds to our MPSS expression profile. For example, expression of OsNCX2 (*OsCAX1a*) was found to be high in all the tissues analyzed by Kamiya et al. (2005), which corresponds to the MPSS data presented in our study. Expression of OsNCX10 (*OsCAX1b*) was high in case of root (NDR) in MPSS data, which also corresponds to the observations by Kamiya et al. (2005). Expression of *OsNCX5* (*OsCAX1c*) was observed only in case of leaf blade (Kamiya et al. 2005), similarly in MPSS data, its expression was observed in case of leaf only. These observations indicate towards possible function of various NCX genes in different tissues and developmental stages. Further, we identified orthologous genes from Arabidopsis and rice to correlate their expression profiles (Additional file 4: Table S3). Interestingly, *AtNCX2* and its ortholog *OsNCX1*; and *AtNCX6* and its ortholog *OsNCX2* were found to have high expression in leaf, root and inflorescence tissues (Fig. 4a and b).

### Microarray-based expression analysis of NCX family genes in Arabidopsis and rice

**Fig. 5:**
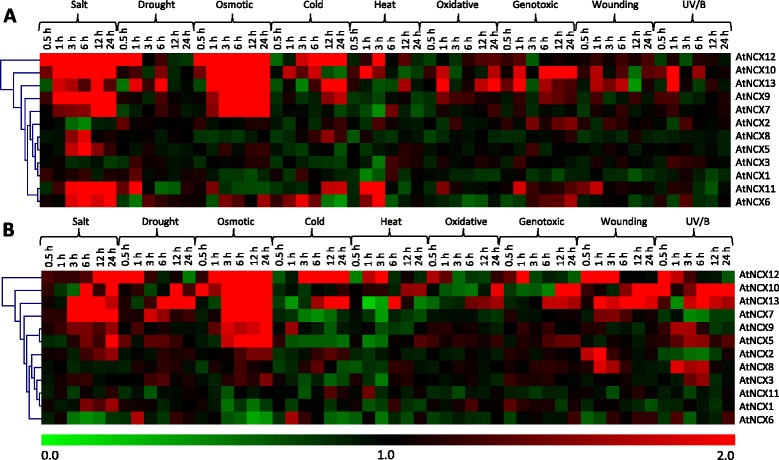
Microarray-based expression profile of Arabidopsis NCX genes under various abiotic stress conditions. Heat maps show the fold changes in expression of Arabidopsis NCX genes in **a** root and **b** shoot tissues under different abiotic stress conditions such as salt, drought, osmotic, cold, heat, oxidative, genotoxic, wounding and UV/B stress. Heat maps were generated using microarray data obtained from AtGenExpress. Microarray data was obtained for different time points for different stresses viz 0.5 h, 1 h, 3 h, 6 h, 12 h and 24 h for both root and shoot tissues and analyzed with respect to control. Relative signal values are represented by color bar shown at the bottom of heat map; thereby green color representing downregulation, black signifies no change in expression and red shows upregulation

**Fig. 6:**
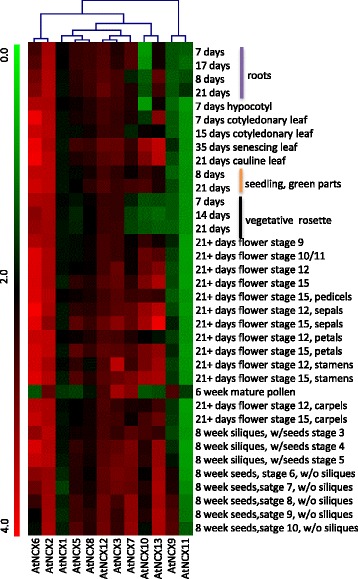
Expression analysis of Arabidopsis NCX genes at different developmental stages using microarray data. Heat map and hierarchical cluster displays differential expression profile of NCX genes across various developmental stages. Color bar at the left represents log_10_ expression values; thereby green color representing lowest expression levels, black medium expression and red signifies highest expression level. Developmental stages used for expression profiling are mentioned on the right side of the heat map

NCX proteins can alter cytosolic Ca^2+^ levels, which is an important second messenger during various physiological and developmental signals. However, most of the plant NCX proteins are uncharacterized and their physiological function remains to be studied. Studying the expression profiling of the uncharacterized genes can provide important clues regarding their function (Mustafiz et al. 2011; Tripathi et al. 2015). Therefore, to gain preliminary insight into the possible function of plant NCX proteins in stress response and during development, we have exploited publicly available microarray data for Arabidopsis and rice. Arabidopsis Microarray data in response to different abiotic stresses (Fig. 5a and b) and different developmental and reproductive stages (Fig. 6) was retrieved from AtGenExpress (http://jsp.weigelworld.org/expviz/expviz.jsp). As shown in Fig. 5, *AtNCX7, AtNCX9, AtNCX10, AtNCX12* and *AtNCX13,* exhibited upregulation under salt and osmotic stress conditions in both root and shoot. Expression of *AtNCX2* in root largely remained unchanged in response to all the stresses. While in shoot, its expression was upregulated in response to salt, osmotic, cold and wounding stresses. Previously, Wang et al. (2012) have also shown its induction in response to salt, ABA, cold stress and heat stress. *AtNCX1* and *AtNCX3* are the only two genes which did not show any significant change in expression in roots. Expression of *AtNCX6* and *AtNCX11* was found to be upregulated in response to salt, cold and heat stresses in roots, while in shoots their expression was largely unaltered. The expression data for *AtNCX4* (At1g55720) was not available in the microarray database and hence could not be included in expression analysis under different stresses.

We also performed the expression profiling of NCX genes during different vegetative and reproductive stages using Microarray data (Fig. 6). Microarray data contain more tissues and developmental stages as compared to MPSS data. Largely, expression profile of NCX genes obtained from microarray data corroborate with MPSS data. For instance, *AtNCX2* and *AtNCX6* were found to be highly expressed in all the developmental stages, which is in agreement to their expression profile observed in MPSS data. On the other hand expression of *AtNCX11* was low at almost all the developmental stages. Specific high transcript of *AtNCX9* in stage 8, 9 and 10 of seed development, indicates toward its particular role in seed development.

**Fig. 7:**
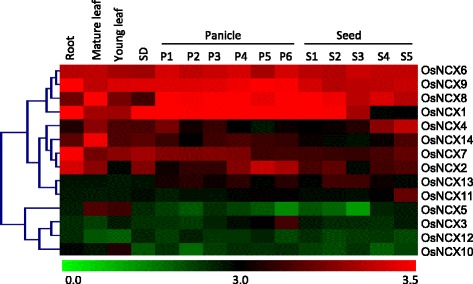
Expression analysis of rice NCX genes at different developmental stages using microarray data. Heat map and hierarchical clustering displays differential expression profile of rice NCX genes across various developmental stages. Color bar at the bottom represents log_2_ expression values, thereby green color representing lowest expression levels, black medium expression and red signifies highest expression level. Developmental stages used for expression analysis are mentioned on the top of each column. Various stages used for the expression profiling (from left to right in the heat map) are: Roots of 7- day old seedling, Mature leaf (collected before pollination), young leaf, SD-7-day old seedling, P1 (0–3 cm panicle), P2 (3–5 cm panicle), P3 (5–10 cm panicle), P4 (10–15 cm panicle), P5 (15–22 cm panicle), P6 (22–30 cm panicle), S1 (0–2 DAP), S2 (3–4 DAP), S3 (5–10 DAP), S4 (11–20 DAP) and S5 (21–29 DAP)

Gene Expression Omnibus (GEO) database of the NCBI (http://www.ncbi.nlm.nih.gov/geo/) was used to obtain transcriptional data of NCX genes under different developmental stages of rice (Fig. 7). Based on the studies by Itoh et al. (2005) and information from Oryzabase (http://www.shigen.nig.ac.jp/rice/oryzabase/top/top.jsp), rice panicle and seed developmental stages were divided into 6 and 5 major categories, respectively. We found that some of the NCX genes like, *OsNCX1, OsNCX6, OsNCX8* and *OsNCX9* were expressed at high level in almost all the developmental stages, suggesting their broad role in plant development. The expression of *OsNCX10* and *OsNCX12* was low in all the developmental stages. Expression of *OsNCX2* and *OsNCX7* was also found to be intermediate in all the developmental stages. Earlier using GUS-reporter assay the expression of *OsCAX1a* (named as *OsNCX2* in the present study) has been found in stomata, trichomes and vascular cells (Kamiya et al. 2006). Our development stage specific microarray data also complements the expression profile of CAX genes as reported previously (Kamiya et al. 2005), which shows high transcript levels of *OsCAX1a* (*OsNCX2)*, *OsCAX2* (*OsNCX7*) and *OsCAX3* (*OsNCX9*) while *OsCAX1b* (*OsNCX10*) and *OsCAX1c* (*OsNCX5*) show very low transcript level in different plant tissues. On comparing the expression profiles of orthologous genes in Arabidopsis and rice (orthologous genes are shown in Additional file 4: Table S3), it was found that *AtNCX2* and its ortholog *OsNCX1*; *AtNCX3* and its ortholog *OsNCX6*; and *AtNCX6* and its ortholog *OsNCX2* have constitutive expression in all the tissues analyzed in Figs. 6 and 7.

### Expression profiling of rice NCX genes using quantitative real time PCR under various abiotic stress and Ca^2+^ stimuli conditions

**Fig. 8:**
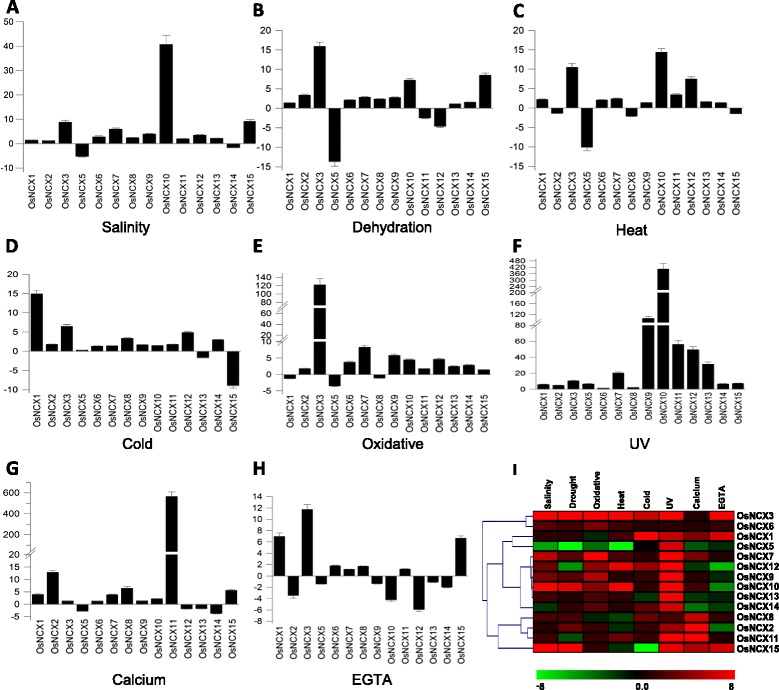
Expression profiling of rice NCX genes under different abiotic stresses using qRT-PCR. Bar graphs show fold change in expression of rice NCX genes under various abiotic stresses, viz. **a** salinity, **b** dehydration, **c** heat, **d** cold, **e** oxidative (MV), **f** UV-B, **g** calcium (calcium chloride) and **h** calcium chelator EGTA **i** Heat map generated from the above qRT-PCR data representing the expression profile of rice NCX genes under different abiotic stresses and calcium and EGTA treatment. Color bars at the base represent fold change in expression; green, black and red colors represent downregulated, unaltered and upregulated expression of genes relative to the untreated control, respectively

Calcium is one of the most important secondary messengers in plant system and its role in various adverse environmental conditions like biotic and abiotic stresses has been well studied. NCX proteins play a pivotal role in Ca^2+^-homeostasis and hence can potentially participate in the diverse physiological processes involving Ca^2+^ as the second messenger. To examine the role of rice NCX family members in abiotic stress and calcium homeostasis and to validate the expression profile obtained via analysis of microarray-based expression data, we have carried out expression analysis using quantitative real-time reverse transcription-PCR (qRT-PCR) in rice. The relative transcript abundance of all the OsNCX genes under various abiotic stresses *viz.* salinity, dehydration, oxidative, heat, cold and UV stress and treatments with calcium and EGTA has been presented as bar graphs and heat maps (Fig. 8a–i). Previous reports have shown that stress related genes are often differentially expressed in rice (Kumari et al. 2009). Expression of *OsNCX3*, *OsNCX9* and *OsNCX10* was either upregulated or unaltered under all the stresses imposed in present study which suggests that these NCX proteins might have some important role in response to abiotic stress. In contrast, expression of *OsNCX5* was repressed under all the stresses except under UV stress. Interestingly, we found that relative transcript abundance of all the NCX genes was upregulated in response to UV stress. Particularly, *OsNCX9, OsNCX10, OsNCX11* and *OsNCX12* were highly up regulated by 100, 300, 56 and 50 fold, respectively in response to UV. *OsNCX3* seems to be highly responsive to oxidative stress as it showed 122-fold upregulation in response to MV. In response to cold stress transcript level of *OsNCX1* was specifically high, suggesting it might have some role in maintaining calcium level during cold stress. The expression of *OsNCX3, OsNCX10* and *OsNCX15* was predominantly up-regulated in response to salinity and dehydration stress. Transcript levels of *OsNCX4* were very low, thus we were not able to detect it under any of the conditions tested in the present study.

Sodium calcium exchangers are involved in intracellular regulation of calcium levels. If Ca^2+^ concentration is low inside the cell, NCX proteins help the cell to intake calcium from outside and vice-versa if calcium concentration is high inside the cell. To study the calcium mediated- regulation of NCX genes at transcriptional level, we examined the transcript abundance of *OsNCX* genes in a moderately stress-sensitive rice cultivar – IR64, in response to calcium and its chelator (EGTA). The transcript level of most of the NCX genes was upregulated in response to exogenous calcium, with some exceptions like *OsNCX5, OsNCX12*, *OsNCX13* and *OsNCX14,* which were downregulated under Ca^2+^ treatment. *OsNCX11* was highly upregulated in response to calcium. Interestingly, EGTA treatment repressed the transcript level of *OsNCX* genes. However, expression of *OsNCX1, OsNCX3*, *OsNCX6*, *OsNCX7*, *OsNCX11* and *OsNCX15* was upregulated in response to EGTA. *OsNCX12* showed extremely low expression in response to both calcium and EGTA implying its restricted role in calcium transportation across or inside the cell. This data indicates that NCX genes are under tight control of intracellular calcium levels. However, it is also apparent from the expression profile obtained in our study that expression pattern of NCX genes is different in response to NaCl and calcium treatments, suggesting their unique role in specific conditions. Further characterization of putative OsNCX family members, at both molecular and chemical level, needs to be carried out in order to ascertain their role in specific abiotic stress conditions.

## Conclusions

Plants require various transporters for exchange of different ions. NCX proteins are one of them which maintain the intracellular and extracellular Ca^2+^ concentrations. However, not much has been studied about plant NCX proteins, and their entire repertoire remains to be discovered. The current study has comprehensively identified genes encoding putative NCX proteins in Arabidopsis, rice, *Brachypodium*, foxtail millet, poplar and potato based on NCX domain. We have also proposed new nomenclature to NCX family members of Arabidopsis and rice, which could be helpful for future studies. The differential expression pattern of NCX members under various developmental stages and stress conditions suggest their role in associated physiological processes. qRT-PCR based expression analysis of rice NCX family members has suggested many new genes involved in abiotic stress response and future studies are needed to validate their function. Taken together, this study has imparted new insights into the putative functions of NCX proteins in plants. Detailed functional characterization of individual NCX family member would be a future challenge and it could help in understanding the role(s) of NCX proteins in various aspects of the life cycle of plants.

## Methods

### Identification of Na^+^/Ca^2+^ exchanger proteins from various dicots and monocots

In order to identify Sodium/calcium exchanger (NCX) proteins in Arabidopsis and rice, hidden markov model (HMM) search was performed in TAIR 10 and RGAP 7 databases, respectively with an e-value cut off 0.001. Similarly, HMM search was performed to identify NCX proteins in *Brachypodium distachyon*, *Setaria italica*, *Populus trichocarpa* and *Solanum tuberosum* using phytozome version 10.1 database (http://phytozome.jgi.doe.gov/pz/portal.html; Goodstein et al. 2012). All the putative NCX proteins identified using HMM search were subjected to Pfam (http://Pfam.sanger.ac.uk/) and InterProScan (http://www.ebi.ac.uk/Tools/pfa/iprscan5/) analysis to verify the presence of NCX domains. BLASTn search program was used to determine the position of each NCX gene on Arabidopsis and rice chromosomes available at TAIR (ver.10) and RGAP (ver.7.0), respectively. For nomenclature prefix “At” or “Os” for *Arabidopsisthaliana* and *Oryza sativa*, respectively was added followed by NCX and numbered according to its position from top to bottom on the respective chromosome. Alternatively spliced forms were represented by Arabic numbers after “.” sign. For identification of orthologous proteins of *O. sativa* NCXs in *B. distachyon* and *S. italica*; and for *A. thaliana* NCX proteins in *P. trichocarpa* and *S. tuberosum*, Blastp search was performed in respective proteome database in phytozome version 10.1.

### Prediction of putative transmembrane topology of NCX proteins from rice and Arabidopsis

The putative transmembrane topology for longest ORF of each of the NCX proteins was predicted with the help of PROTTER version 1.0 (Omasits et al. 2014; http://wlab.ethz.ch/protter/start/).

### Phylogenetic analysis

Multiple sequence alignment was performed using ClustalW2 (Larkin et al. 2007) and phylogenetic tree for NCX proteins was plotted using MEGA6 software (Tamura et al. 2013) using Neighbour-joining method (Saitou and Nei 1987) with 1000 bootstrap replicates. Rest of the parameters used in MEGA 6 were set to default.

### Expression analysis using MPSS database

Expression data from MPSS tags was retrieved from the Arabidopsis and rice MPSS project websites (http://mpss.udel.edu). The signature was considered to be significant if it uniquely identifies an individual gene and shows perfect match (100 % identity over 100 % length of the tag). The normalized abundance (tags per million, tpm) of these signatures for a given gene in a given library represents a quantitative estimate of expression of that gene. Descriptions of the MPSS libraries of *A. thaliana* and *O. sativa* are provided in Additional file 5: Table S4 and Additional file 6: Table S5, respectively. Heatmaps were generated using expression values obtained from MPSS database for respective NCX genes with the help MeV software (http://www.tm4.org/mev.html, Eisen et al. 1998).

### Expression analysis using microarray data

Expression profile of Arabidopsis NCX genes was analyzed using microarray data generated through AtGenExpress (https://www.arabidopsis.org/portals/expression/microarray/ATGenExpress.jsp) under different abiotic stress conditions such as, salt, drought, osmotic, cold, heat, oxidative, genotoxic, wounding and UV/B stress (Kilian et al. 2007) and various developmental stages (Schmid et al. 2005). Microarray data was obtained for different stresses at different time points viz 0.5 h, 1 h, 3 h, 6 h, 12 h and 24 h for both root and shoot tissues. Fold change at transcript level of different genes under stress was calculated with respect to their controls. For the developmental stage data, Affymetrix values were log10 transformed, heat maps generated and hierarchical clustering done using the MeV software package (Eisen et al. 1998). For microarray analysis of rice NCX genes, Affymetrix GeneChip rice genome arrays (http://www.ncbi.nlm.nih.gov/geo/; Gene Expression Omnibus platform accession nos. GSE6893) was used. The Affymetrix values were log2 transformed and heatmaps generated using TIGR MeV software (Eisen et al. 1998). Microarray dataset in rice is described as: Roots of 7- day old seedling, Mature leaf (collected before pollination), young leaf, SD-7-day old seedling, P1 (0-3 cm panicle), P2 (3–5 cm panicle), P3 (5–10 cm panicle), P4 (10–15 cm panicle), P5 (15–22 cm panicle), P6 (22–30 cm panicle), S1 (0–2 DAP), S2 (3–4 DAP), S3 (5–10 DAP), S4 (11–20 DAP) and S5 (21–29 DAP).

### Plant material and stress treatment for qRT-PCR analysis

Seeds of IR64 rice cultivar were germinated in hydroponic system. The seedlings were grown under control conditions in Yoshida medium (Yoshida et al. 1972) in a growth chamber at 28 ± 2 °C and 16 h photoperiod. After 10 days, various stress treatments were given to seedlings viz. salinity stress (200 mM NaCl), dehydration (air dry), oxidative stress (10 μM methyl viologen), heat stress (42 °C), cold stress (4 °C), UV stress (UV-B exposure), calcium (100 mM CaCl_2_) or calcium chelator (10 mM EGTA). The duration of all the stress treatments was 6 h and untreated seedlings were taken as the experimental control. After 6 h, shoots were cut, weighed and frozen in liquid nitrogen for further use.

Total RNA was isolated from the frozen stressed and control shoot samples using RaFlex™ solution I and solution II (Bangalore Genei, India) according to the manufacturer’s protocol. Total RNA was used for first strand cDNA synthesis using RevertAidTM RNAse H minus cDNA synthesis kit as per manufacturer’s protocol (Fermentas Life Sciences, USA). Primers were designed from 3′ UTR unique region for each of the rice NCX gene using Primer3 software. Sequences of the primers have been provided as Additional file 7: Table S6. For qRT- PCR, the reaction mixture comprised 5 μl of 10 times diluted cDNA, 12.5 μl of 2X SYBR Green PCR Master Mix (Applied Biosystems, USA) and 200 nM of each gene specific primer in a final volume of 25 μl reaction mixture in 48 well optical reaction plates (Applied Biosystems, USA). Applied Biosystems Step OneTM Real time PCR machine was used to perform qRT-PCR. The PCR conditions for all the genes were kept same i.e. 10 min at 95 °C and 35 cycles of 15 s at 95 °C, 30 s at 55 °C and 30 s at 72 °C. Melt curve analysis was performed for each experiment for testing the specificity of the amplification. The relative expression value was calculated by using REST 2009 software (Vandesompele et al. 2002). At least two biological replicates and three technical replicates were analyzed for qRT-PCR.

## Additional files

Additional file 1: Figure S1. Phylogenetic relationships among Arabidopsis and rice NCX proteins. Unrooted tree was generated using MEGA6 software using the Neighbor-joining method with 1000 bootstrap replicates. NCX proteins are categorized into five different clades depending upon the relative sequence homology of each member of the clades. Each clade is denoted by different color shading. Additional file 2: Table S1. List of orthologous proteins of Arabidopsis NCX proteins in poplar and potato and rice NCX proteins in *Brachypodium* and foxtail millet. Orthologous proteins were identified in Phytozome 10.1 against database of respective species using Blastp search function. Additional file 3: Table S2. Position of CAX, NCX and EF hand domains in NCX proteins of *Brachypodium*, foxtail millet, poplar and potato. Additional file 4: Table S3. Orthologous proteins of Arabidopsis NCXs in rice alongwith locus IDs of their respective genes. Additional file 5: Tables S4. Description of Arabidopsis MPSS libraries. Additional file 6: Tables S5. Description of rice MPSS libraries. Additional file 7: Table S6. List of primers used for qRT-PCR analysis and their sequence, and the expected amplicon size.

## Abbreviations

NCX: Sodium/Calcium exchanger
CAX: Calcium/proton exchangers
EF: EF hand
EGTA: Ethylene glycol tetraacetic acid
MV: Methyl viologen
UV: Ultra violet
TMS: Transmembrane segments
