# Supplementary material for: Genome-wide investigation and expression analysis of Sodium/Calcium exchanger gene family in rice and Arabidopsis
Source: Rice (N Y). 2015 Jul 2;8:21. doi: 10.1186/s12284-015-0054-5 (PMC4488139; doi:10.1186/s12284-015-0054-5)
Supplement: Additional file 1: Figure S1. — Phylogenetic relationships among Arabidopsis and rice NCX proteins. Unrooted tree was generated using MEGA6 software using the Neighbor-joining method with 1000 bootstrap replicates. NCX proteins are categorized into five different clades depending upon the relative sequence homology of each member of the clades. Each clade is denoted by different color shading. [file 12284_2015_54_MOESM1_ESM.pptx]

## Slide 1
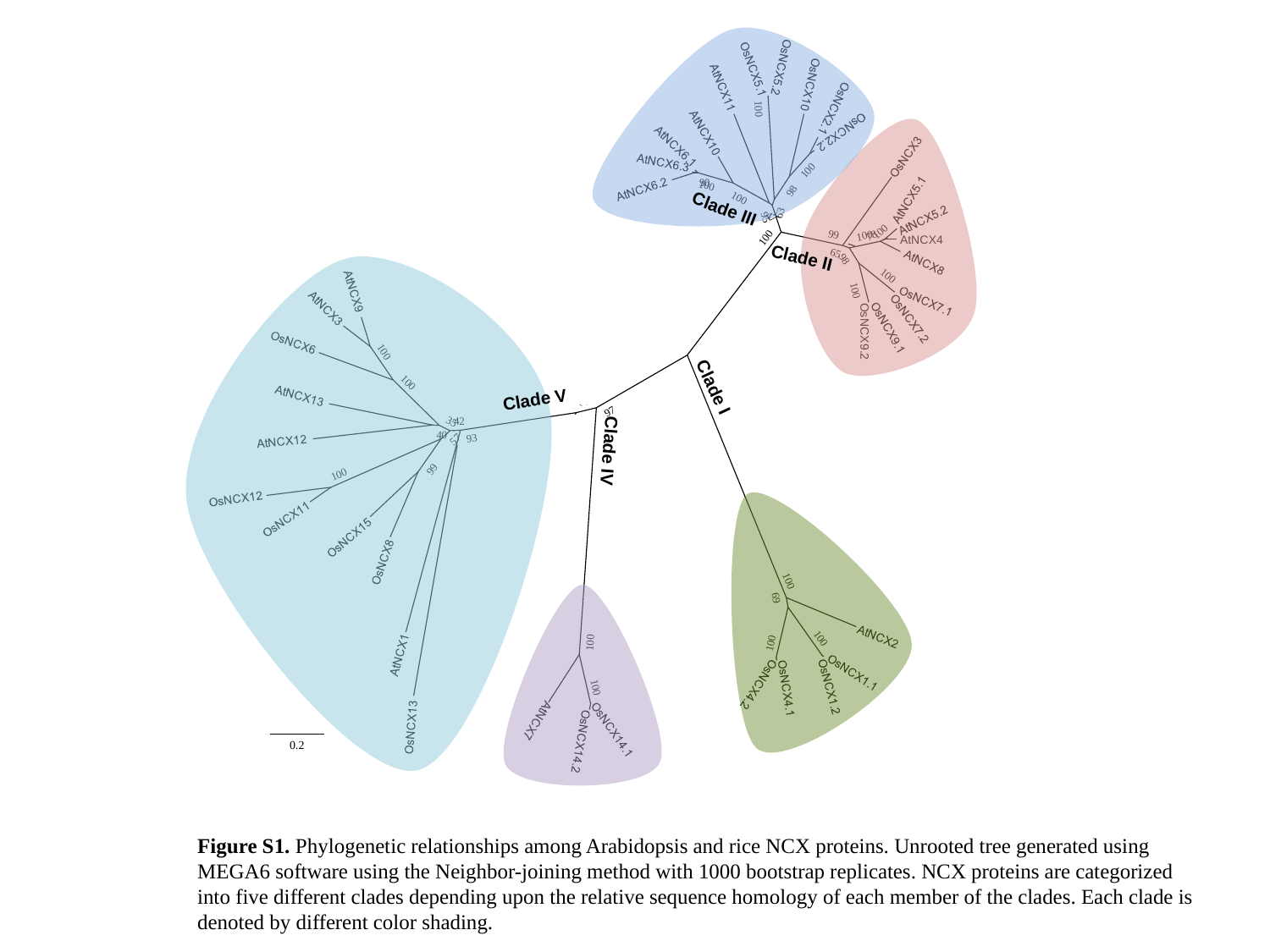

Clade III
Clade II
Clade I
Clade V
Clade IV
Figure S1. Phylogenetic relationships among Arabidopsis and rice NCX proteins. Unrooted tree generated using MEGA6 software using the Neighbor-joining method with 1000 bootstrap replicates. NCX proteins are categorized into five different clades depending upon the relative sequence homology of each member of the clades. Each clade is denoted by different color shading.
