# Supplementary material for: Genome-wide investigation and expression analysis of Sodium/Calcium exchanger gene family in rice and Arabidopsis
Source: Rice (N Y). 2015 Jul 2;8:21. doi: 10.1186/s12284-015-0054-5 (PMC4488139; doi:10.1186/s12284-015-0054-5)
Supplement: Additional file 2: Table S1. — List of orthologous proteins of Arabidopsis NCX proteins in poplar and potato and rice NCX proteins in Brachypodium and foxtail millet. Orthologous proteins were identified in Phytozome 10.1 against database of respective species using Blastp search function. [file 12284_2015_54_MOESM2_ESM.docx]

**Additional file 2: Table S1**. List of proteins orthologous to Arabidopsis NCX proteins in poplar and potato; as well as of those orthologous to rice NCX proteins in Brachypodium and foxtail millet. Orthologous proteins were identified using Phytozome 10.1 *via* Blastp search function against database of the respective species.

| **Arabidopsis NCX proteins** | **Orthologs in Poplar (*Populus trichocarpa*)** | **bit score** | **E-value** |
| --- | --- | --- | --- |
| AtNCX1 | Potri.013G025100.1 | 472.2 | 4.20E-162 |
| AtNCX2 | Potri.001G375500.1 | 707.6 | 0 |
| AtNCX3 | Potri.003G066900.1 | 752.7 | 0 |
| AtNCX4 | Potri.001G469800.2 | 575.1 | 0 |
| AtNCX5.1 | Potri.001G469800.1 | 612.8 | 0 |
| AtNCX5.2 | Potri.001G469800.1 | 612.8 | 0 |
| AtNCX6.1 | Potri.006G099900.5 | 471.9 | 5.60E-162 |
| AtNCX6.2 | Potri.016G115500.3 | 332.4 | 1.40E-109 |
| AtNCX6.3 | Potri.006G099900.3 | 467.6 | 2.80E-160 |
| AtNCX7 | Potri.014G128600.1 | 732.3 | 0 |
| AtNCX8 | Potri.001G469800.2 | 639.4 | 0 |
| AtNCX9 | Potri.003G066900.1 | 646 | 0 |
| AtNCX10 | Potri.016G115500.1 | 487.3 | 1.80E-168 |
| AtNCX11 | Potri.016G115500.4 | 463.4 | 1.20E-159 |
| AtNCX12 | Potri.013G065900.1 | 367.1 | 1.10E-119 |
| AtNCX13 | Potri.019G040200.1 | 505.8 | 4.70E-172 |
| **Arabidopsis NCX proteins** | **Orthologs in Potato (*Solanum tuberosum*)** | **bit score** | **E-value** |
| AtNCX1 | PGSC0003DMT400073651 | 463 | 1.50E-158 |
| AtNCX2 | PGSC0003DMT400032376 | 729.9 | 0 |
| AtNCX3 | PGSC0003DMT400054444 | 782.3 | 0 |
| AtNCX4 | PGSC0003DMT400023738 | 533.5 | 0 |
| AtNCX5.1 | PGSC0003DMT400023738 | 553.9 | 0 |
| AtNCX5.2 | PGSC0003DMT400023738 | 553.9 | 0 |
| AtNCX6.1 | PGSC0003DMT400030772 | 469.5 | 9.20E-162 |
| AtNCX6.2 | PGSC0003DMT400030772 | 335.1 | 3.00E-110 |
| AtNCX6.3 | PGSC0003DMT400030772 | 465.7 | 5.10E-160 |
| AtNCX7 | No hit |  |  |
| AtNCX8 | PGSC0003DMT400023737 | 578.2 | 0 |
| AtNCX9 | PGSC0003DMT400054444 | 655.2 | 0 |
| AtNCX10 | PGSC0003DMT400030772 | 480.3 | 5.20E-166 |
| AtNCX11 | PGSC0003DMT400030772 | 444.1 | 8.00E-152 |
| AtNCX12 | PGSC0003DMT400071681 | 340.5 | 4.70E-108 |
| AtNCX13 | PGSC0003DMT400062404 | 471.5 | 4.30E-160 |
| **Rice NCX proteins** | **Orthologs in Brachypodium (*Brachypodium distachyon*)** | **bit score** | **E-value** |
| OsNCX1.1 | Bradi2g06830 | 885.9 | 0 |
| OsNCX1.2 | Bradi2g06830 | 774.6 | 0 |
| OsNCX2.1 | Bradi2g41087.1 | 635.6 | 0 |
| OsNCX2.2 | Bradi2g41087.1 | 446.8 | 2.60E-154 |
| OsNCX3 | Bradi3g03544.1 | 451.4 | 5.50E-156 |
| OsNCX4.1 | Bradi3g09350.1 | 864 | 0 |
| OsNCX4.2 | Bradi3g09350.1 | 693.7 | 0 |
| OsNCX5.1 | Bradi3g11066.1 | 469.5 | 2.60E-162 |
| OsNCX5.2 | Bradi3g11066.1 | 469.5 | 2.60E-162 |
| OsNCX6 | Bradi1g72680.1 | 1004.6 | 0 |
| OsNCX7.1 | Bradi1g60440.1 | 712.6 | 0 |
| OsNCX7.2 | Bradi1g60440.1 | 717.6 | 0 |
| OsNCX8 | Bradi1g13240.1 | 458.8 | 5.10E-154 |
| OsNCX9.1 | Bradi5g24387.2 | 741.1 | 0 |
| OsNCX9.2 | Bradi5g24387.2 | 741.1 | 0 |
| OsNCX10 | Bradi5g20177.1 | 567 | 0 |
| OsNCX11 | Bradi3g27220.1 | 434.1 | 2.90E-144 |
| OsNCX12 | Bradi3g27220.1 | 227.3 | 4.20E-68 |
| OsNCX13 | Bradi4g42875.1 | 113.2 | 3.30E-28 |
| OsNCX14.1 | Bradi4g11030.1 | 854 | 0 |
| OsNCX14.2 | Bradi4g11030.1 | 670.6 | 0 |
| OsNCX15 | Bradi1g62270.1 | 318.5 | 1.10E-99 |
| **Rice NCX proteins** | **Orthologs in Foxtail millet (*Setaria italica*)** | **bit score** | **E-value** |
| OsNCX1.1 | Si000723m | 847.8 | 0 |
| OsNCX1.2 | Si000723m | 741.5 | 0 |
| OsNCX2.1 | Si001368m | 635.6 | 0 |
| OsNCX2.2 | Si001368m | 440.3 | 6.20E-152 |
| OsNCX3 | Si019435m | 417.5 | 2.50E-143 |
| OsNCX4.1 | Si016772m | 867.1 | 0 |
| OsNCX4.2 | Si016772m | 697.6 | 0 |
| OsNCX5.1 | Si017853m | 412.5 | 1.20E-141 |
| OsNCX5.2 | Si017853m | 412.5 | 1.20E-141 |
| OsNCX6 | Si034644m | 1018.5 | 0 |
| OsNCX7.1 | Si035077m | 708.8 | 0 |
| OsNCX7.2 | Si035077m | 710.3 | 0 |
| OsNCX8 | Si039744m | 468.8 | 3.40E-158 |
| OsNCX9.1 | Si022169m | 743.4 | 0 |
| OsNCX9.2 | Si022169m | 743.4 | 0 |
| OsNCX10 | Si021879m | 559.7 | 0 |
| OsNCX11 | Si035502m | 391.3 | 4.40E-129 |
| OsNCX12 | Si035502m | 176.4 | 7.20E-50 |
| OsNCX13 | Si010009m | 147.5 | 2.00E-40 |
| OsNCX14.1 | Si026220m | 799.7 | 0 |
| OsNCX14.2 | Si026220m | 627.5 | 0 |
| OsNCX15 | Si021538m | 338.6 | 3.80E-107 |
