# Supplementary material for: Genome-wide investigation and expression analysis of Sodium/Calcium exchanger gene family in rice and Arabidopsis
Source: Rice (N Y). 2015 Jul 2;8:21. doi: 10.1186/s12284-015-0054-5 (PMC4488139; doi:10.1186/s12284-015-0054-5)
Supplement: Additional file 3: Table S2. — Position of CAX, NCX and EF hand domains in NCX proteins of Brachypodium, foxtail millet, poplar and potato. [file 12284_2015_54_MOESM3_ESM.docx]

**Additional file 3: Table S2.** Position of CAX, NCX and EF hand domains in NCX proteins from Brachypodium, Foxtail millet, Poplar and Potato

| **S.No** | **Protein** | **CAX domain** | **NCX domain** | **EF Hand** |
| --- | --- | --- | --- | --- |
| **Brachypodium (*Brachypodium distachyon*)** | | | | |
| 1 | Bradi1g13240.1 |  | 129-263, 425-565 |  |
| 2 | Bradi1g60440.1 | 71-420 | 98-244, 286-416 |  |
| 3 | Bradi1g62270.1 |  | 120-260, 418-559 |  |
| 4 | Bradi1g72680.1 |  | 150-280, 484-625 |  |
| 5 | Bradi2g06830.1 |  | 441-571 | 307-335, 347-375 |
| 6 | Bradi2g41087.1 | 78-434 | 104-254, 301-429 |  |
| 7 | Bradi3g03544.1 | 100-444 | 120-266, 311-440 |  |
| 8 | Bradi4g42880.1 |  | 4-143 |  |
| 9 | Bradi3g27220.1 |  | 112-251, 420-564 |  |
| 10 | Bradi4g11030.3 |  | 11-60, 218-371 |  |
| 11 | Bradi4g11030.1 |  | 43-204, 362-515 |  |
| 12 | Bradi4g42867.1 |  | 60-189 |  |
| 13 | Bradi5g20177.1 | 79-429 | 102-252, 296-425 |  |
| 14 | Bradi5g20177.2 | 79-429 | 102-252, 296-425 |  |
| 15 | Bradi5g24387.2 | 56-407 | 82-228, 273-403 |  |
| 16 | Bradi3g11066.1 | 50-412 | 99-232, 279-410 |  |
| 17 | Bradi4g42875.1 |  | 60-189,344-483 |  |
| 18 | Bradi3g09350.1 |  | 498-628 | 364-392, 404-432 |
| **Foxtail millet (*Setaria italica*)** | | | | |
| 1 | Si035077m | 180-529 | 207-353, 395-525 |  |
| 2 | Si000723m | 116-466 | 139-289, 333-461 |  |
| 3 | Si010009m |  | 471-601 | 337-365,377-405 |
| 4 | Si001372m | 82-431 | 106-256,106-256 |  |
| 5 | Si034644m |  | 146-276,482-623 |  |
| 6 | Si035502m |  | 15-150,323-463 |  |
| 7 | Si039744m |  | 123-253, 410-550 |  |
| 8 | Si017853m | 1-300 | 2-122,167-298 |  |
| 9 | Si027804m |  | 58-185, 327-466 |  |
| 10 | Si022169m | 57-407 | 82-228, 273-403 |  |
| 11 | Si019435m | 61-406 | 81-227,272-402 |  |
| 12 | Si016772m |  | 431-561 | 293-328, 333-368 |
| 13 | Si026220m |  | 39-200, 358-512 |  |
| 14 | Si021538m |  | 142-278, 449-590 |  |
| 15 | Si001368m | 82-431 | 106-256, 297-426 |  |
| **Poplar (*Populus trichocarpa*)** | | | | |
| 1 | Potri.014G128600.1 |  | 54-215, 367-520 |  |
| 2 | Potri.016G115500.1 | 74-423 | 99-245,290-418 |  |
| 3 | Potri.001G375500.1 |  | 450-580 | 310-338,350 378 |
| 4 | Potri.001G375300.1 |  | 431-563 | 304-332, 344-372 |
| 5 | Potri.001G167200.1 |  | 155-286,466-606 |  |
| 6 | Potri.001G375400.1 |  | 512-644 | (247-275, 287-315) (384-412, 424-452) |
| 7 | Potri.006G099900.1 | 105-454 | 130-276, 321-449 |  |
| 8 | Potri.001G251200.1 | 87-431 | 112-258,297-428 |  |
| 9 | Potri.010G249200.1 | 73-424 | 99-245, 290-420 |  |
| 10 | Potri.003G066900.1 |  | 155-286, 454-594 |  |
| 11 | Potri.013G065800.1 |  | 119-250, 424-564 |  |
| 12 | Potri.019G040300.1 |  | 55-178 |  |
| 13 | Potri.019G040200.1 |  | 121-252, 428-569 |  |
| 14 | Potri.001G469800.1 | 86-438 | 112-258, 304-434 |  |
| 15 | Potri.011G166900.1 | 86-438 | 112-258, 304-434 |  |
| 16 | Potri.013G025100.1 |  | 105-228, 391-530 |  |
| 17 | Potri.013G065900.1 |  | 67-149, 323-462 |  |
| 18 | Potri.009G045800.1 | 89-432 | 114-260, 298-429 |  |
| **Potato (*Solanum tuberosum*)** | | | | |
| 1 | PGSC0003DMP400049838 |  | 107-237,407-546 |  |
| 2 | PGSC0003DMP400016189 | 73-424 | 99-245,290-420 |  |
| 3 | PGSC0003DMP400013720 |  | 99-229,274-430 |  |
| 4 | PGSC0003DMP400022702 |  | 559-691 | (290-318,330 358) (427-455,467-495) |
| 5 | PGSC0003DMP400042017 |  | 42-179, 357-498 |  |
| 6 | PGSC0003DMP400048490 |  | 133-265, 468-607 |  |
| 7 | PGSC0003DMP400026880 |  | 435-566 | 299-327, 339-367 |
| 8 | PGSC0003DMP400021992 |  | 440-572 | 304-332, 344-372 |
| 9 | PGSC0003DMP400054145 | 74-422 | 97-243, 288-418 |  |
| 10 | PGSC0003DMP400020895 | 81-432 | 108-256, 298-427 |  |
| 11 | PGSC0003DMP400036631 |  | 151-283, 489-629 |  |
| 12 | PGSC0003DMP400019866 |  | 4-85, 245-386 |  |
| 13 | PGSC0003DMP400039823 |  | 572-703 | (302-330,343-371) (440-468, 480-508) |
| 14 | PGSC0003DMP400007432 | 53-408 | 79-231,274-402 |  |
| 15 | PGSC0003DMP400016191 | 73-424 | 99-245, 290-420 |  |
| 16 | PGSC0003DMP400016190 | 73-424 | 99-245, 290-420 |  |
