# Supplementary material for: Genome-wide investigation and expression analysis of Sodium/Calcium exchanger gene family in rice and Arabidopsis
Source: Rice (N Y). 2015 Jul 2;8:21. doi: 10.1186/s12284-015-0054-5 (PMC4488139; doi:10.1186/s12284-015-0054-5)
Supplement: Additional file 4: Table S3. — Orthologous proteins of Arabidopsis NCXs in rice alongwith locus IDs of their respective genes. [file 12284_2015_54_MOESM4_ESM.docx]

| **Arabidopsis NCX protein** | **Orthologous proteins in rice** | **Locus ID of respective rice gene** |
| --- | --- | --- |
| AtNCX1 | OsNCX13 | LOC_Os11g05070.1 |
| AtNCX2 | OsNCX1 | LOC_Os01g11414.1 |
| AtNCX3 | OsNCX6 | LOC_Os03g08230.1 |
| AtNCX4 | OsNCX7 | LOC_Os03g27960.2 |
| AtNCX5.1 | OsNCX9 | LOC_Os04g55940.2 |
| AtNCX5.2 | OsNCX9 | LOC_Os04g55940.2 |
| AtNCX6.1 | OsNCX2 | LOC_Os01g37690.1 |
| AtNCX6.2 | OsNCX2 | LOC_Os01g37690.1 |
| AtNCX6.3 | OsNCX2 | LOC_Os01g37690.1 |
| AtNCX7 | OsNCX14 | LOC_Os11g43860.1 |
| AtNCX8 | OsNCX7 | LOC_Os03g27960.2 |
| AtNCX9 | OsNCX6 | LOC_Os03g08230.1 |
| AtNCX10 | OsNCX2 | LOC_Os01g37690.1 |
| AtNCX11 | OsNCX10 | LOC_Os05g51610.1 |
| AtNCX12 | OsNCX8 | LOC_Os03g45370.1 |
| AtNCX13 | OsNCX8 | LOC_Os03g45370.1 |

**Additional file 4: Table S3.** Orthologous proteins of Arabidopsis NCXs in rice along with locus ids of their respective genes
