# Supplementary material for: Genome-wide investigation and expression analysis of Sodium/Calcium exchanger gene family in rice and Arabidopsis
Source: Rice (N Y). 2015 Jul 2;8:21. doi: 10.1186/s12284-015-0054-5 (PMC4488139; doi:10.1186/s12284-015-0054-5)
Supplement: Additional file 5: Tables S4. — Description of Arabidopsis MPSS libraries. [file 12284_2015_54_MOESM5_ESM.docx]

| **Sl. No.** | **Library** | **Description of library** |
| --- | --- | --- |
| 1 | CAF | Callus, hardened tissue that forms to protect the exposed areas of cuttings |
| 2 | CAS | Callus (hardened tissue that forms to protect the exposed areas of cuttings) – actively growing |
| 3 | INF | Inflorescence, part of the plant that consists of flower bearing stalks |
| 4 | INS | Inflorescence – mixed stage, immature buds |
| 5 | AP1 | ap1-10 inflorescence (part of the plant that consists of flower bearing stalks) – mixed stage, immature buds |
| 6 | AP3 | ap3-6 inflorescence (part of the plant that consists of flower bearing stalks) – mixed stage, immature buds |
| 7 | AGM | agamous inflorescence (part of the plant that consists of flower bearing stalks) – mixed stage, immature buds |
| 8 | SAP | sup/ap1 inflorescence – mixed stage, immature buds |
| 9 | S04 | Leaves, 4 hr after salicylic acid treatment |
| 10 | S52 | Leaves, 52 hr after salicylic acid treatment |
| 11 | LEF | Leaves – 21 day, untreated |
| 12 | LES | Leaves – 21 day, untreated |
| 13 | ROF | Root – 21 day, untreated |
| 14 | ROS | Root – 21 day, untreated |
| 15 | GSE | Germinating seedlings |
| 16 | SIF | Silique (Seedpod) – 24 to 48 hr post-fertilization |
| 17 | SIS | Silique (Seedpod) – 24 to 48 hr post-fertilization |

**Additional file 5: Tables S4.** Description of Arabidopsis MPSS libraries
