# Supplementary material for: Genome-wide investigation and expression analysis of Sodium/Calcium exchanger gene family in rice and Arabidopsis
Source: Rice (N Y). 2015 Jul 2;8:21. doi: 10.1186/s12284-015-0054-5 (PMC4488139; doi:10.1186/s12284-015-0054-5)
Supplement: Additional file 6: Tables S5. — Description of rice MPSS libraries. [file 12284_2015_54_MOESM6_ESM.docx]

**Additional file 6: Tables S5.** Description of rice MPSS libraries

| **Sl. No.** | **Library** | **Library description** |
| --- | --- | --- |
| 1 | 9LA | Leaves Replicate A |
| 2 | 9LB | Leaves Replicate B |
| 3 | 9LC | Leaves Replicate C |
| 4 | 9LD | Leaves Replicate D |
| 5 | FLA | F1 Hybrid 60 days Mature Leaf Replicate A |
| 6 | FLB | F1 Hybrid 60 days Mature Leaf Replicate B |
| 7 | FLC | F1 Hybrid 60 days Mature Leaf Replicate C |
| 8 | FLD | F1 Hybrid 60 days Mature Leaf Replicate D |
| 9 | NCL | 14 days – Young leaves stressed in 4°C cold for 24 h |
| 10 | NCR | 14 days – Young roots stressed in 4°C cold for 24 h |
| 11 | NDL | 14 days – Young leaves stressed in drought for 5 days |
| 12 | NLA | 60 days – Mature Leaves – Replicate A |
| 13 | NLB | 60 days – Mature Leaves – Replicate B |
| 14 | NLC | 60 days – Mature Leaves – Replicate C |
| 15 | NLD | 60 days – Mature Leaves – Replicate D |
| 16 | NSL | 14 days – Young leaves stressed in 250 mM NaCl for 24 h |
| 17 | NYL | NPO – Mature Pollen, 14 days – Young leaves |
| 18 | PLA | rice leaf, beet armyworm damaged, 24 hr |
| 19 | PLC | rice leaf, mechanical damaged, 24 hr |
| 20 | PLW | rice leaf, water weevil damaged, 24 hr |
| 21 | 9ME | Meristematic Tissue |
| 22 | NME | 60 days – Crown vegetative meristematic tissue |
| 23 | 9RO | Roots |
| 24 | 9RR | Roots – Replicate |
| 25 | NDR | 14 days – Young roots stressed in drought for 5 days |
| 26 | FME | F1 Hybrid 60 days Meristematic tissue |
| 27 | FRO | F1 Hybrid 60 days Mature Root |
| 28 | FRR | F1 Hybrid 60days Mature Root-Replicate |
| 29 | NYR | 14 days – Young Roots |
| 30 | NRA | 60 days – Mature Roots – Replicate A |
| 31 | NRB | 60 days – Mature Roots – Replicate B |
| 32 | NSR | 14 days – Young roots stressed in 250 mM NaCl for 24 h |
| 33 | NCA | 35 days – Callus |
| 34 | NIP | 90 days – Immature panicle |
| 35 | NOS | Ovary and mature stigma |
| 36 | NPO | Mature Pollen |
| 37 | NST | 60 days – Stem |
| 38 | NGD | 10 days – Germinating seedlings grown in dark |
| 39 | NGS | 3 days – Germinating seed |
| 40 | PSC | rice developing seeds, 6 days old cypress high milling (99–1710) |
| 41 | PSI | rice developing seeds,6 days old, Il pumbyeo – High Taste |
| 42 | PSL | rice developing seeds, 6 days old, LaGrue-Low Milling |
| 43 | PSN | rice developing seed, 6 days old, Nipponbare-Grain quality control |
| 44 | PSY | rice developing seeds, 6 days old |
