# Supplementary material for: Genome-wide investigation and expression analysis of Sodium/Calcium exchanger gene family in rice and Arabidopsis
Source: Rice (N Y). 2015 Jul 2;8:21. doi: 10.1186/s12284-015-0054-5 (PMC4488139; doi:10.1186/s12284-015-0054-5)
Supplement: Additional file 7: Table S6. — List of primers used for qRT-PCR analysis and their sequence, and the expected amplicon size. [file 12284_2015_54_MOESM7_ESM.docx]

| **Sl. No.** | **Gene Name** | **Forward primer sequence (5’-3’)**   \|  \| \| --- \| | **Reverse primer sequence (5’-3’)** | **Amplicon size (bp)** |
| --- | --- | --- | --- | --- | --- |
| 1 | OsNCX1 | GCTGTGCATTGTAACTTGTG | CTTGCTTGGTTACTTTCAGG | 118 |
| 2 | OsNCX2 | GTCTCCTGAACAAAACATGG | CATGATGATCTGTCTTGCTG | 100 |
| 3 | OsNCX3 | ATGTTTGGGATACCATTTTG | ACAGTTGTTAAGAGGCTTGC | 101 |
| 4 | OsNCX4 | AATCTAAGAGGACGTGTTGC | GCCAACAAAGGAACTACAAC | 111 |
| 5 | OsNCX5 | TTAATCAGCAACAACAGCAG | AACACCAAAGCAATCAAAAC | 102 |
| 6 | OsNCX6 | ATTGCCTGGCTTGAGTTAG | TAAAGACAGGCATGGATAGG | 93 |
| 7 | OsNCX7 | GTGGAATGATAAGGGATGAG | TGTGAGATACTGCTGACAGG | 120 |
| 8 | OsNCX8 | ACGTAGCCACGTACTACACC | TGTTAGCTAAAGGCTTGTGAG | 107 |
| 9 | OsNCX9 | AAGTCTCCTTGCATAGTTGC | AATTAAGCCCCAAAAATGAC | 100 |
| 10 | OsNCX10 | GTACGTACATGCGCCTAAC | CTCGATCCTCTTCTCTTGTG | 108 |
| 11 | OsNCX11 | TGGTGATGTCCAGGAGAG | AAGTTCATACCGGTCCAAG | 94 |
| 12 | OsNCX12 | CAGAGACGAAGTTCTACGG | GCAAGTTTCCTGACATGC | 116 |
| 13 | OsNCX13 | CATACAGCCCTTCACACTG | AGCAAGATCTCCCCTCTG | 112 |
| 14 | OsNCX14 | ACATGGCCATTTTTACACAC | TTTTCTCAGGCAGAAAAGC | 156 |
| 15 | OsNCX15 | TGATGATGCTAGATTGGTTG | ATGCATTATTTCATTGCATC | 181 |
| 16 | eEF-1α | TTTCACTCTTGGTGTGAAGCAGAT | GACTTCCTTCACGATTTCATCGTAA | 103 |

**Additional file 7: Table S6.** List of primers used for qRT-PCR analysis, their sequence and the expected amplicon size.
